# Supplementary material for: Modelling alcohol consumption patterns to enable policy impact assessment
Source: PLoS One. 2025 Dec 1;20(12):e0327264. doi: 10.1371/journal.pone.0327264 (PMC12668553; doi:10.1371/journal.pone.0327264)
Supplement: S1 File — (DOCX) [file pone.0327264.s001.docx]

S1. National Health Survey items

Table A provides the tags of the questions on alcohol use that were used from the National Health Survey for estimating the alcohol model. Complete information on all questions can be obtained using the codebooks listed by CBS [1].

**Table A.** **Questions on alcohol use that were used from the National Health Survey.**

| **Time period** | **Tags of questions used for submodel 1 (drinking) and**  **submodel 2 (number of alcoholic beverages per week)** | **Tags of questions used for submodel 3 (heavy drinking)** |
| --- | --- | --- |
| 2004-2009 | v24a; v27; v28; v29; v30; v31; v32 | v25; v26 |
| 2010 & 2011 | sralchol1; wannralc; ddwalc; dagddwk; dagddwk; wendalc; dagwend; glaswend | meer6alc; hoev6alc |
| 2012 & 2013 | alcoholsr1; ddwkdag; ddwkglas; wenddag; wendglas; | alcmeer4; alcmeer6 |
| 2014-2018 | gezvrgalcohol; gezvrgalcoholweekdagen; gezvrgalcoholweekglazen; gezvrgalcoholweekenddagen; gezvrgalcoholweekendglazen | gezvrgalcohol4glazen; gezvrgalcohol6glazen |

# References

1. CBS. Gezondheidsenquête vanaf 2014: Centraal Bureau voor de Statistiek (CBS) / Statistics Netherlands; 2024 [Available from: <https://www.cbs.nl/nl-nl/onze-diensten/methoden/onderzoeksomschrijvingen/korte-onderzoeksomschrijvingen/gezondheidsenquete-vanaf-2014>.
