# Supplementary material for: Modelling alcohol consumption patterns to enable policy impact assessment
Source: PLoS One. 2025 Dec 1;20(12):e0327264. doi: 10.1371/journal.pone.0327264 (PMC12668553; doi:10.1371/journal.pone.0327264)
Supplement: S2 File — (DOCX) [file pone.0327264.s002.docx]

S2. Data baseline characteristics

Table A provides baseline characteristics of the National Health Survey data used in the analysis [1].

**Table A.** **Data baseline characteristics.** Baseline characteristics on demographics and alcohol use (weighted for Dutch population) in the National Health Survey for Dutch adult men and women in 2008-2022. NABW: number of alcoholic beverages per week.

|  | **N** | **mean age [years]  (sd)** | **education [%]** | | | **drinking [%]** | **mean  NABW (sd)** | **excessive  drinking [%]** | **heavy  drinking [%]** |
| --- | --- | --- | --- | --- | --- | --- | --- | --- | --- |
|  |  |  | **low** | **middle** | **high** |  |  |  |  |
| *men* | 48,182 | 48.6 (17.5) | 26.6 | 40.1 | 33.3 | 82.6 | 9.4 (12.4) | 10.2 | 12.6 |
| *women* | 51,430 | 49.6 (18.1) | 32.6 | 37.1 | 30.3 | 68.1 | 4.6 (7.9) | 6.6 | 6.7 |

# References

1. CBS, RIVM, Trimbos-instituut. Gezondheidsenquête (National Health Survey). In: CBS, RIVM, Trimbos-instituut, editors. 2008–2022.
