# Supplementary material for: Modelling alcohol consumption patterns to enable policy impact assessment
Source: PLoS One. 2025 Dec 1;20(12):e0327264. doi: 10.1371/journal.pone.0327264 (PMC12668553; doi:10.1371/journal.pone.0327264)
Supplement: S3 File — (DOCX) [file pone.0327264.s003.docx]

S1. National Health Survey items

Fig A shows the prevalence of drinking for Dutch adults over calendar time. The trend breaks occur between the years 2009 and 2010 (changed questions and repositioning of questions), 2011 and 2012 (extra question for definition change for heavy drinking for women) and 2013 and 2014 (changed questions and repositioning of questions) [1-3]. It can be observed from the figure that these trend breaks have little impact on the trend in the drinking prevalence.





**Fig A.** **Prevalence of drinking (%) for Dutch adults over calendar time in the National Health Survey.**

# References

1. Cobben F, Roels J, Wong FY, Buelens B, Banning R, van den Brakel J, et al. Methodologisch onderzoek bij het herontwerp van de Gezondheidsenquête 2010. Centraal Bureau voor de Statistiek (CBS) / Statistics Netherlands; 2012.

2. Wong FY, Roels J, van den Brakel J. Analyse methodebreuken Gezondheidsenquête bij het herontwerp Persoonsenquête. Centraal Bureau voor de Statistiek (CBS) / Statistics Netherlands; 2011.

3. Morren M, Willems R. Gezondheidsenquête 2014: een analyse van de methodebreuk. Centraal Bureau voor de Statistiek (CBS) / Statistics Netherlands; 2015.
