# Supplementary material for: Modelling alcohol consumption patterns to enable policy impact assessment
Source: PLoS One. 2025 Dec 1;20(12):e0327264. doi: 10.1371/journal.pone.0327264 (PMC12668553; doi:10.1371/journal.pone.0327264)
Supplement: S4 File — (DOCX) [file pone.0327264.s004.docx]

S4. Calibration procedure

A calibration procedure yielded scaling parameters for the negative binomial distribution predicting excessive and heavy drinking. The calibration was performed through numerical optimization with the Nelder-Mead algorithm [1]. The goal function that is minimized in this procedure is the following:

$$\sum_{s} ssqd(\alpha)\cdot(1+\lambda_{\mu}^{\alpha}\cdot\left| \alpha_{\mu_{s}}-1 \right|+\lambda_{\Theta}^{\alpha}\cdot\left| \alpha_{\Theta_{s}}-1 \right|) , \quad\quad(1)$$

with scaling parameters $\alpha$, penalty terms $\lambda$, sex s, and where

$$ssqd(\alpha)=\sum_{s} {(\frac{p_{{fit}_{s|\mu, \Theta}}(\alpha) - p_{{data}_{s}}}{p_{{data}_{s}}})}^{2} ,\quad\quad(2)$$

The sum of squared differences, $ssqd$, squares the relative difference between the prevalence of excessive drinking or heavy drinking in the fit $p_{{fit}_{s}}$ and the prevalence of excessive drinking or heavy drinking in the data $p_{{data}_{s}}$. The prevalence of excessive drinking or heavy drinking in the fit, $p_{{fit}_{s}}$, follows from the negative binomial distribution which incorporates scaling parameters $\alpha$ ($\kappa$ and $\lambda$ in equation 4), given the constant negative binomial parameters $\mu$, $\Theta$ and a population distribution of age, education and calendar time.

The parameters $\lambda^{\alpha}$ describe the penalty terms for the corresponding scaling parameters $\alpha$. The penalty term also scales with the $ssqd$. These penalty terms were incorporated such that the optimization procedure would not lead to values of $\alpha$ which are unnecessarily high and were thus used to tune the optimization procedure. The values that were used in the calibration procedure were $\lambda_{\mu}^{\alpha}=0.5$ and $\lambda_{\Theta}^{\alpha}=0.1$ for both excessive and heavy drinking, as we found it more important to find correct estimates for the mean NABW than for its dispersion.

# References

1. R Core Team. R: A language and environment for statistical computing. R

Foundation for Statistical Computing, Vienna, Austria. 2024.
