# Supplementary material for: Modelling alcohol consumption patterns to enable policy impact assessment
Source: PLoS One. 2025 Dec 1;20(12):e0327264. doi: 10.1371/journal.pone.0327264 (PMC12668553; doi:10.1371/journal.pone.0327264)
Supplement: S5 File — (DOCX) [file pone.0327264.s005.docx]

S5. Model parameters

The following sections list the parameters of three submodels. The text box below provides extra information on B-spline parameters

*B-spline parameters*

The age relationships in submodel 1 and 2 are represented using B-splines. A B-spline (basis spline) is a flexible way to model complex, nonlinear, relationships in data. A B-spline is defined by a set of base functions, which are combined to construct the B-spline curve. For this, each basis function is weighted, such that the contribution to the B-spline is determined. Hyperparameters control how the B-spline is constructed from the base functions. These hyperparameters are the degree of freedom and the knots (points to divide the B-spline in intervals). In this study’s B-spline implementation, equally spaced knots were used. More information on B-splines can be obtained elsewhere, for instance in the documentation of the “splines2” package which was used in this study to estimate the splines [1].

# Submodel 1

Table A shows all parameter values for submodel 1, predicting drinking or not drinking. The parameters correspond to equation 1 in the main article.

**Table A. Parameter values for submodel 1.**

| **Parameter** | **value** |
| --- | --- |
| hyperparameter for degree for intercept term | 3 |
| hyperparameter for lower boundary knot for intercept term | 12.5 |
| hyperparameter for upper boundary knot for intercept term | 87.5 |
| hyperparameter for number of internal knots for intercept term | 5 |
| hyperparameter for degree for time dependent term | 2 |
| hyperparameter for lower boundary knot for time dependent term | 12.5 |
| hyperparameter for upper boundary knot for time dependent term | 87.5 |
| hyperparameter for number of internal knots for time dependent term | 0 |
| base function 1 weight for the log odds at calendar time = 0 (intercept), for men with low education | 135.0826 |
| base function 2 weight for the log odds at calendar time = 0 (intercept), for men with low education | 139.2366 |
| base function 3 weight for the log odds at calendar time = 0 (intercept), for men with low education | 132.6202 |
| base function 4 weight for the log odds at calendar time = 0 (intercept), for men with low education | 116.2945 |
| base function 5 weight for the log odds at calendar time = 0 (intercept), for men with low education | 92.46563 |
| base function 6 weight for the log odds at calendar time = 0 (intercept), for men with low education | 61.77986 |
| base function 7 weight for the log odds at calendar time = 0 (intercept), for men with low education | 22.99943 |
| base function 8 weight for the log odds at calendar time = 0 (intercept), for men with low education | -8.12353 |
| base function 9 weight for the log odds at calendar time = 0 (intercept), for men with low education | -24.898 |
| base function 1 weight for the time dependent term of the log odds, for men with low education | -0.06904 |
| base function 2 weight for the time dependent term of the log odds, for men with low education | -0.06146 |
| base function 3 weight for the time dependent term of the log odds, for men with low education | 0.01257 |
| base function 1 weight for the log odds at calendar time = 0 (intercept), for men with middle education | 151.0498 |
| base function 2 weight for the log odds at calendar time = 0 (intercept), for men with middle education | 149.003 |
| base function 3 weight for the log odds at calendar time = 0 (intercept), for men with middle education | 131.5339 |
| base function 4 weight for the log odds at calendar time = 0 (intercept), for men with middle education | 106.285 |
| base function 5 weight for the log odds at calendar time = 0 (intercept), for men with middle education | 81.90784 |
| base function 6 weight for the log odds at calendar time = 0 (intercept), for men with middle education | 57.93849 |
| base function 7 weight for the log odds at calendar time = 0 (intercept), for men with middle education | 34.2404 |
| base function 8 weight for the log odds at calendar time = 0 (intercept), for men with middle education | 17.90218 |
| base function 9 weight for the log odds at calendar time = 0 (intercept), for men with middle education | 10.22293 |
| base function 1 weight for the time dependent term of the log odds, for men with middle education | -0.07713 |
| base function 2 weight for the time dependent term of the log odds, for men with middle education | -0.03874 |
| base function 3 weight for the time dependent term of the log odds, for men with middle education | -0.0047 |
| base function 1 weight for the log odds at calendar time = 0 (intercept), for men with high education | -4.79558 |
| base function 2 weight for the log odds at calendar time = 0 (intercept), for men with high education | 33.56094 |
| base function 3 weight for the log odds at calendar time = 0 (intercept), for men with high education | 90.34616 |
| base function 4 weight for the log odds at calendar time = 0 (intercept), for men with high education | 131.8062 |
| base function 5 weight for the log odds at calendar time = 0 (intercept), for men with high education | 135.4715 |
| base function 6 weight for the log odds at calendar time = 0 (intercept), for men with high education | 99.10927 |
| base function 7 weight for the log odds at calendar time = 0 (intercept), for men with high education | 22.69452 |
| base function 8 weight for the log odds at calendar time = 0 (intercept), for men with high education | -54.8814 |
| base function 9 weight for the log odds at calendar time = 0 (intercept), for men with high education | -100.055 |
| base function 1 weight for the time dependent term of the log odds, for men with high education | 0.00086 |
| base function 2 weight for the time dependent term of the log odds, for men with high education | -0.15127 |
| base function 3 weight for the time dependent term of the log odds, for men with high education | 0.05033 |
| base function 1 weight for the log odds at calendar time = 0 (intercept), for women with low education | 46.83798 |
| base function 2 weight for the log odds at calendar time = 0 (intercept), for women with low education | 72.47528 |
| base function 3 weight for the log odds at calendar time = 0 (intercept), for women with low education | 97.83613 |
| base function 4 weight for the log odds at calendar time = 0 (intercept), for women with low education | 112.8425 |
| base function 5 weight for the log odds at calendar time = 0 (intercept), for women with low education | 100.7385 |
| base function 6 weight for the log odds at calendar time = 0 (intercept), for women with low education | 61.57245 |
| base function 7 weight for the log odds at calendar time = 0 (intercept), for women with low education | -6.00808 |
| base function 8 weight for the log odds at calendar time = 0 (intercept), for women with low education | -68.8816 |
| base function 9 weight for the log odds at calendar time = 0 (intercept), for women with low education | -105.067 |
| base function 1 weight for the time dependent term of the log odds, for women with low education | -0.0255 |
| base function 2 weight for the time dependent term of the log odds, for women with low education | -0.10853 |
| base function 3 weight for the time dependent term of the log odds, for women with low education | 0.05194 |
| base function 1 weight for the log odds at calendar time = 0 (intercept), for women with middle education | 79.96782 |
| base function 2 weight for the log odds at calendar time = 0 (intercept), for women with middle education | 94.31702 |
| base function 3 weight for the log odds at calendar time = 0 (intercept), for women with middle education | 101.7471 |
| base function 4 weight for the log odds at calendar time = 0 (intercept), for women with middle education | 100.8332 |
| base function 5 weight for the log odds at calendar time = 0 (intercept), for women with middle education | 85.89858 |
| base function 6 weight for the log odds at calendar time = 0 (intercept), for women with middle education | 55.73243 |
| base function 7 weight for the log odds at calendar time = 0 (intercept), for women with middle education | 10.7791 |
| base function 8 weight for the log odds at calendar time = 0 (intercept), for women with middle education | -30.1499 |
| base function 9 weight for the log odds at calendar time = 0 (intercept), for women with middle education | -52.4794 |
| base function 1 weight for the time dependent term of the log odds, for women with middle education | -0.04194 |
| base function 2 weight for the time dependent term of the log odds, for women with middle education | -0.07391 |
| base function 3 weight for the time dependent term of the log odds, for women with middle education | 0.02609 |
| base function 1 weight for the log odds at calendar time = 0 (intercept), for women with high education | -87.7434 |
| base function 2 weight for the log odds at calendar time = 0 (intercept), for women with high education | -35.1173 |
| base function 3 weight for the log odds at calendar time = 0 (intercept), for women with high education | 43.47259 |
| base function 4 weight for the log odds at calendar time = 0 (intercept), for women with high education | 113.4135 |
| base function 5 weight for the log odds at calendar time = 0 (intercept), for women with high education | 136.943 |
| base function 6 weight for the log odds at calendar time = 0 (intercept), for women with high education | 112.296 |
| base function 7 weight for the log odds at calendar time = 0 (intercept), for women with high education | 39.90019 |
| base function 8 weight for the log odds at calendar time = 0 (intercept), for women with high education | -41.2502 |
| base function 9 weight for the log odds at calendar time = 0 (intercept), for women with high education | -89.3889 |
| base function 1 weight for the time dependent term of the log odds, for women with high education | 0.04187 |
| base function 2 weight for the time dependent term of the log odds, for women with high education | -0.16965 |
| base function 3 weight for the time dependent term of the log odds, for women with high education | 0.04451 |

# Submodel 2

Table B shows all parameter values for submodel 2, predicting the number of alcoholic beverages per week (NABW) and excessive drinking. The parameter values correspond to equations 2-4 in the main article.

**Table B. Parameter values for submodel 2.**

| **parameter** | **value** |
| --- | --- |
| hyperparameter for degree for intercept term | 3 |
| hyperparameter for lower boundary knot for intercept term | 12.5 |
| hyperparameter for upper boundary knot for intercept term | 87.5 |
| hyperparameter for number of internal knots for intercept term | 4 |
| hyperparameter for degree for time dependent term | 2 |
| hyperparameter for lower boundary knot for time dependent term | 12.5 |
| hyperparameter for upper boundary knot for time dependent term | 87.5 |
| hyperparameter for number of internal knots for time dependent term | 0 |
| base function 1 weight for log(mu) where calendar time = 0 (intercept), for men with low education | 76.49612 |
| base function 2 weight for log(mu) where calendar time = 0 (intercept), for men with low education | 79.5036 |
| base function 3 weight for log(mu) where calendar time = 0 (intercept), for men with low education | 78.91824 |
| base function 4 weight for log(mu) where calendar time = 0 (intercept), for men with low education | 69.166 |
| base function 5 weight for log(mu) where calendar time = 0 (intercept), for men with low education | 49.41813 |
| base function 6 weight for log(mu) where calendar time = 0 (intercept), for men with low education | 20.76825 |
| base function 7 weight for log(mu) where calendar time = 0 (intercept), for men with low education | -5.69774 |
| base function 8 weight for log(mu) where calendar time = 0 (intercept), for men with low education | -19.8846 |
| base function 1 weight for log(mu) where calendar time = 0 (intercept), for men with middle education | 32.05955 |
| base function 2 weight for log(mu) where calendar time = 0 (intercept), for men with middle education | 39.41363 |
| base function 3 weight for log(mu) where calendar time = 0 (intercept), for men with middle education | 46.71377 |
| base function 4 weight for log(mu) where calendar time = 0 (intercept), for men with middle education | 52.17915 |
| base function 5 weight for log(mu) where calendar time = 0 (intercept), for men with middle education | 51.94022 |
| base function 6 weight for log(mu) where calendar time = 0 (intercept), for men with middle education | 45.06714 |
| base function 7 weight for log(mu) where calendar time = 0 (intercept), for men with middle education | 36.1914 |
| base function 8 weight for log(mu) where calendar time = 0 (intercept), for men with middle education | 30.45404 |
| base function 1 weight for log(mu) where calendar time = 0 (intercept), for men with high education | 23.06281 |
| base function 2 weight for log(mu) where calendar time = 0 (intercept), for men with high education | 34.45928 |
| base function 3 weight for log(mu) where calendar time = 0 (intercept), for men with high education | 49.83765 |
| base function 4 weight for log(mu) where calendar time = 0 (intercept), for men with high education | 65.23818 |
| base function 5 weight for log(mu) where calendar time = 0 (intercept), for men with high education | 72.8964 |
| base function 6 weight for log(mu) where calendar time = 0 (intercept), for men with high education | 71.79322 |
| base function 7 weight for log(mu) where calendar time = 0 (intercept), for men with high education | 64.78644 |
| base function 8 weight for log(mu) where calendar time = 0 (intercept), for men with high education | 59.95598 |
| base function 1 weight for time dependent term of log(mu), for men with low education | -0.03742 |
| base function 2 weight for time dependent term of log(mu), for men with low education | -0.04266 |
| base function 3 weight for time dependent term of log(mu), for men with low education | 0.010912 |
| base function 1 weight for time dependent term of log(mu), for men with middle education | -0.01555 |
| base function 2 weight for time dependent term of log(mu), for men with middle education | -0.03425 |
| base function 3 weight for time dependent term of log(mu), for men with middle education | -0.01413 |
| base function 1 weight for time dependent term of log(mu), for men with high education | -0.01089 |
| base function 2 weight for time dependent term of log(mu), for men with high education | -0.04623 |
| base function 3 weight for time dependent term of log(mu), for men with high education | -0.02866 |
| base function 1 weight for log(mu) where calendar time = 0 (intercept), for women with low education | 7.157536 |
| base function 2 weight for log(mu) where calendar time = 0 (intercept), for women with low education | 22.20231 |
| base function 3 weight for log(mu) where calendar time = 0 (intercept), for women with low education | 45.87319 |
| base function 4 weight for log(mu) where calendar time = 0 (intercept), for women with low education | 70.58469 |
| base function 5 weight for log(mu) where calendar time = 0 (intercept), for women with low education | 83.0385 |
| base function 6 weight for log(mu) where calendar time = 0 (intercept), for women with low education | 82.89792 |
| base function 7 weight for log(mu) where calendar time = 0 (intercept), for women with low education | 74.4608 |
| base function 8 weight for log(mu) where calendar time = 0 (intercept), for women with low education | 67.98115 |
| base function 1 weight for log(mu) where calendar time = 0 (intercept), for women with middle education | 22.73712 |
| base function 2 weight for log(mu) where calendar time = 0 (intercept), for women with middle education | 38.07109 |
| base function 3 weight for log(mu) where calendar time = 0 (intercept), for women with middle education | 58.20867 |
| base function 4 weight for log(mu) where calendar time = 0 (intercept), for women with middle education | 78.25627 |
| base function 5 weight for log(mu) where calendar time = 0 (intercept), for women with middle education | 85.6263 |
| base function 6 weight for log(mu) where calendar time = 0 (intercept), for women with middle education | 80.16649 |
| base function 7 weight for log(mu) where calendar time = 0 (intercept), for women with middle education | 67.29516 |
| base function 8 weight for log(mu) where calendar time = 0 (intercept), for women with middle education | 58.73693 |
| base function 1 weight for log(mu) where calendar time = 0 (intercept), for women with high education | -85.2196 |
| base function 2 weight for log(mu) where calendar time = 0 (intercept), for women with high education | -38.4157 |
| base function 3 weight for log(mu) where calendar time = 0 (intercept), for women with high education | 35.50539 |
| base function 4 weight for log(mu) where calendar time = 0 (intercept), for women with high education | 101.3717 |
| base function 5 weight for log(mu) where calendar time = 0 (intercept), for women with high education | 119.761 |
| base function 6 weight for log(mu) where calendar time = 0 (intercept), for women with high education | 90.5106 |
| base function 7 weight for log(mu) where calendar time = 0 (intercept), for women with high education | 39.16136 |
| base function 8 weight for log(mu) where calendar time = 0 (intercept), for women with high education | 5.472482 |
| base function 1 weight for time dependent term of log(mu), for women with low education | -0.00292 |
| base function 2 weight for time dependent term of log(mu), for women with low education | -0.05588 |
| base function 3 weight for time dependent term of log(mu), for women with low education | -0.03299 |
| base function 1 weight for time dependent term of log(mu), for women with middle education | -0.01118 |
| base function 2 weight for time dependent term of log(mu), for women with middle education | -0.05926 |
| base function 3 weight for time dependent term of log(mu), for women with middle education | -0.02836 |
| base function 1 weight for time dependent term of log(mu), for women with high education | 0.042883 |
| base function 2 weight for time dependent term of log(mu), for women with high education | -0.12643 |
| base function 3 weight for time dependent term of log(mu), for women with high education | -0.00175 |
| theta parameter for men | 0.89513 |
| theta parameter for women | 0.69345 |
| excessive drinking scale parameter for mu for men | 0.93033 |
| excessive drinking scale parameter for theta for men | 1.30455 |
| heavy drinking scale parameter for mu for men | 0.93705 |
| heavy drinking scale parameter for theta for men | 1.15797 |
| excessive drinking scale parameter for mu for women | 0.85677 |
| excessive drinking scale parameter for theta for women | 0.91542 |
| heavy drinking scale parameter for mu for women | 0.90884 |
| heavy drinking scale parameter for theta for women | 1.22275 |
| trend break coefficient for before t=2010 | 0.055282 |
| trend break coefficient for before t=2012 | -0.11174 |
| trend break coefficient for before t=2014 | -0.12117 |
| trend break coefficient for before t=2010 | -0.01745 |
| trend break coefficient for before t=2012 | -0.06711 |
| trend break coefficient for before t=2014 | -0.22024 |

# Submodel 3

Table C shows all parameter values for submodel 3, predicting heavy drinking. The parameter values correspond to equations 5-9 in the main article.

**Table C. Parameter values for submodel 3.**

| **parameter** | **value** |
| --- | --- |
| intercept | -105.551 |
| trend break coefficient for before t=2012 | 0.22885 |
| trend break coefficient for before t=2010 | -0.11841 |
| trend break coefficient for before t=2014 | 0.85772 |
| coefficient for women | 0.72677 |
| coefficient for middle education | -0.02841 |
| coefficient for high education | -0.13114 |
| linear coefficient for age | 0.94605 |
| quadratic coefficient for age | -0.01597 |
| cubic coefficient for age | -0.00001 |
| calendar time coefficient | 0.051 |
| NABW coefficient | 2.83638 |
| coefficient for interaction between time and age (linear) | -0.0005 |
| coefficient for interaction between time and age (quadratic) | 0.00001 |
| coefficient for interaction between NABW and time | -0.00132 |
| coefficient for interaction between NABW and age | -0.00064 |
| coefficient for interaction between NABW and women | 0.05922 |
| coefficient for interaction between NABW and middle education | 0.01668 |
| coefficient for interaction between NABW and high education | 0.04498 |
| coefficient for interaction between age and women | -0.00984 |
| coefficient for interaction between age and middle education | -0.01006 |
| coefficient for interaction between age and high education | -0.02384 |
| trend break coefficient for women before t=2012 | -1.72775 |
| trend break coefficient for women before t=2010 | 0.14007 |
| trend break coefficient for women before t=2014 | -0.3026 |

# References

1. Wang W, Yan J. Shape-Restricted Regression Splines with R Package splines2. Journal of Data Science. 2021;19(3):498–517.
