# Supplementary material for: Modelling alcohol consumption patterns to enable policy impact assessment
Source: PLoS One. 2025 Dec 1;20(12):e0327264. doi: 10.1371/journal.pone.0327264 (PMC12668553; doi:10.1371/journal.pone.0327264)
Supplement: S6 File — (DOCX) [file pone.0327264.s006.docx]

S6. Results for submodel 1

Fig A shows the estimates of the first submodel, which predicts drinking (NABW=0).


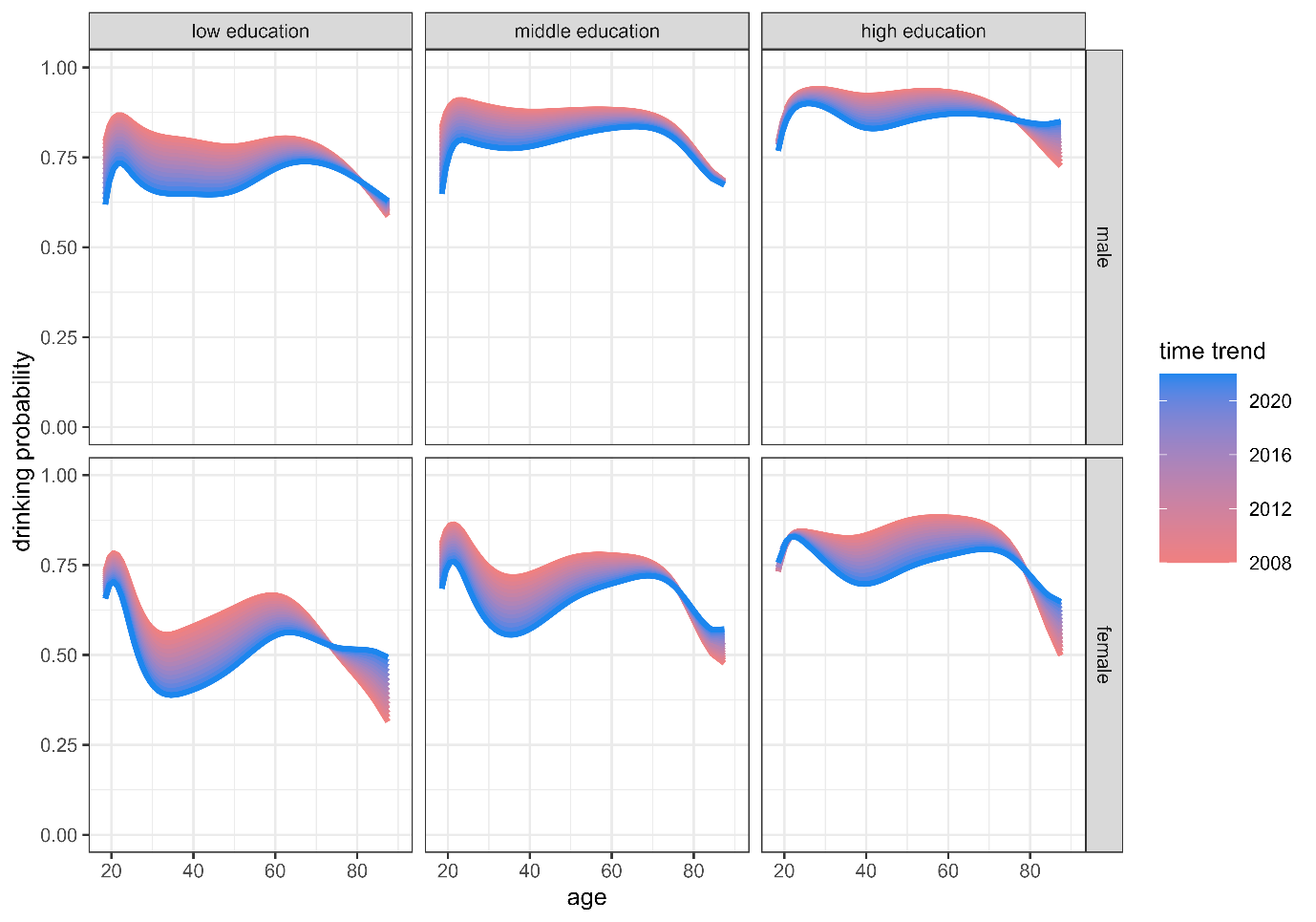


**Fig A. Results for submodel 1.** Predicted drinking probability for adults over age and by sex and education. Colour gradients represent the time trends (utter red: 2008, utter blue: 2022).
