# Supplementary material for: Modelling alcohol consumption patterns to enable policy impact assessment
Source: PLoS One. 2025 Dec 1;20(12):e0327264. doi: 10.1371/journal.pone.0327264 (PMC12668553; doi:10.1371/journal.pone.0327264)
Supplement: Attachment — Submitted filename: Response to reviewers.docx [file pone.0327264.s008.docx]

# Response to reviewers

First of all, we would like to thank the reviewers for their time and comments on the manuscript. We have edited the manuscript to address their concerns. Our revisions to the text are recorded using Track Changes in MS Word. Our point-by-point responses (regular text) to the reviewers’ comments (*italic* text) are shown below. Associated changes in the manuscript are added below our response for each point. We are confident that we have addressed all concerns and that our manuscript is now suitable for publication in PLOS One.

# Reviewer 1

*Introduction - Quantify the Dutch disease burden attributable to excessive and heavy drinking. Add statistics to address the urgency of policy evaluation.*

We agree that this is relevant background information. We now specify the Dutch disease burden to (general) alcohol use in the introduction.

For example, in the Netherlands, it is estimated that 3% of all disability adjusted life years (DALYs) are attributed alcohol use (VZInfo, 2024).

*Methods - The paper states the National Health Survey uses "weighting variables" it doesn’t include details. Add a table detailing weighting methodology and its effect on prevalence estimates.*

The weighting methodology of the National Health Survey is complex, but described in detail in other publications. We now added new references to all publications describing this weighting methodology. The survey weights are required to make the survey representative for the Dutch population, and thus improves prevalence estimates.

The population-level outcomes related to alcohol use from the survey are representative of the Dutch population via the use of supplied weighting variables. Detailed methodological description on the weighing variables is provided in elsewhere by Statistics Netherlands (Boonstra, 2025; Bruggink & van Herten, 2014; CBS, 2005, 2025a, 2025b, 2025c).

*Methods- consider adding Sensitivity Analysis for Trend Breaks.*

The trend breaks are acknowledged and analyzed by the data supplier, see Cobben et al. (2012); Morren and Willems (2015); and Wong et al. (2011). In line with this, we feel that the trend breaks should be adjusted for in the model. To add a sensitivity analysis for the trend breaks, we would have to create a scenario that (to some extent) neglects the trend breaks, which we think is not in line with evidence on the impact of the trend breaks. In addition, our method of adjusting for the trend breaks aligns with the approach of the data supplier by estimating intercepts per trend break period. Therefore, our approach to handling trend breaks in the National Health Survey data ensures that our analysis is compatible with other analyses on this aspect of the data. Because of these reasons, we did not include a sensitivity analysis for the trend breaks.

*Methods - The paper removes individuals with "inconsistent" heavy drinking status (0.4%). This introduces selection bias. Acknowledge it.*

We agree with the reviewer that this must be acknowledged and added this to the discussion.

Next, we removed inconsistent data for the heavy drinking submodel. Although the inconsistent data accounted for only a small fraction (0.4%) of the total, removing them may have induced selection bias.

*Outline data needs for real-world monitoring of model predictions. create an early warning system for policy adjustments that can flag when things start to go off course. Include clear thresholds so you know when it’s time to step in and adjust policies or ramp up interventions.*

We interpret this remark as an indication of potential applications for our model, for which the proposed idea of real-world monitoring is indeed very relevant. Regarding data needs for real world monitoring: the National Health Survey data that was used in our study is sufficient for monitoring, as the data is yearly updated. It has already been used to monitor prevention policies, see e.g. Figure 7 in Boer et al. (2022). This model also has already been applied to monitor prevention policies for the Dutch government, by evaluating whether current prevention measures are sufficient to stay below targets for the prevalences of excessive and heavy drinking in 2040. The idea of an early warning monitoring role with thresholds is hence attractive and in line with previous use. However, defining these thresholds is a societal and/or political aim. We added a statement on earlier application of the model for monitoring prevention policies to the closing statement in the discussion. It is relevant to regularly update estimates of the model’s time trends with data, and to keep comparing real-world data with model predictions to monitor societal trends in alcohol.

However, this study’s alcohol model can already be applied to monitor alcohol use in The Netherlands by evaluating (future) trends of excessive and heavy drinking, as well as by calculating the impact of interventions (Eykelenboom et al., 2024)

# Reviewer 2

*Thank you for the opportunity to review this paper. The authors have undertaken a modeling study to develop predictive models of alcohol consumption patterns including weekly consumption, excessive consumption, and heavy episodic drinking using a representative sample of the Dutch population. Below I include minor comments to help clarify some elements in the manuscript.*

*Lines 78:81: The sentence beginning “Models that predict…” appears to be missing a word as the sentence is difficult to follow.*

We acknowledge that the sentence was difficult to follow. We revised the corresponding section and hope that it is now clearer.

Several existing models predict alcohol use for the general population and are applied in policy evaluation (Brennan et al., 2015; Britton et al., 2015; Koenig et al., 2020; OECD, 2024; Probst et al., 2023; Purshouse et al., 2010). Some of these studies modelled alcohol consumption as a categorical variable (e.g. abstention, moderate consumption, heavy consumption) (Probst et al., 2023; Purshouse et al., 2010). However, the effects of interventions on alcohol use are most often measured through a change in the number of beverages consumed (Fellbaum et al., 2023; Mun et al., 2023; Platt et al., 2016; Tanner-Smith & Risser, 2016). Hence, in a model where alcohol consumption is treated as a categorical variable, any change in the number of beverages should be reflected in the transition probability to another category. Therefore, these models are limited in incorporating interventions. To align an alcohol model with the effect size of interventions, alcohol consumption ideally should be modelled using a continuous or discrete distribution representing the amount of alcohol consumed in more detail.

*There are several places in the manuscript where the authors refer to incorrect figures. For example, Line 135 includes a reference to Figure 2 as a histogram, but this is actually Figure 3.*

Indeed, figures 2 and 3 were switched. All references now refer to the right figures and tables.

*Line 136: Please include the specific version of R used for the analysis as the software does update and functions are sometimes deprecated.*

We included the version of R in the manuscript.

All (statistical) analyses were performed in R version 4.4.2, using RStudio (R Core Team, 2024).

# Reviewer 3

*Thank you for the opportunity to review this manuscript. This study proposes a new alcohol consumption predictive model based on data from The Netherlands. This study would be beneficial for the policy measure. The manuscript was well written. The methods were well designed. There are a few small points that might improve the manuscript:*

*- Line 95: Please give examples for number of alcoholic beverages. Is this equal to standard drink? Are both a can of beer and a bottle of wine counted as one alcoholic beverage, even their alcohol contents are different?*

We agree with the reviewer that an explanation on alcoholic beverages is helpful. An alcoholic beverage is indeed assumed to be equal to a standard drink, containing 10 grams of pure alcohol and ensuring comparability across different beverages. This is now explained in the corresponding section.

The amount of alcohol was assessed in the survey by asking the number of alcoholic beverages on a typical day of consuming alcohol. An alcoholic beverage is assumed to be equivalent to a standard drink, which is a way to ensure comparability across different beverages. The definition of a standard drink varies between countries (Furtwængler & de Visser, 2013; Gezondheidsraad, 2015). In the Netherlands, it is a drink that contains 10 grams of pure alcohol (Gezondheidsraad, 2015). Based on the variables in the data, individual alcohol consumption was calculated and defined through the number of alcoholic beverages per (typical) week (NABW).

*- Line 106-107: Please clarify the levels of education used in this study. Which levels are consider low, middle and high?*

We specified each level of education in the corresponding section.

For education, the lowest level aggregates people with intermediate secondary education or less and basic vocational education, the medium level aggregates higher secondary and intermediate vocational education, and the highest level applies to persons with higher vocational education or university.

*- Line 107 “year”: I think that “year” would indicate survey year. If so, please add detail for clarity.*

“Year” actually indicates the *decimal* year from the moment of filling out the survey. We added this explanation to the corresponding section.

Next to variables on (patterns of) alcohol use, age (by 1 year), sex (male, female), year (expressed as the decimal year in which a respondent completed the survey, further denoted as calendar time), and education (low, middle, high) were extracted from the dataset.

*- Line 361-366: Please explain in more details on how the missing data was handled.*

We added the specification that missing data records were removed in the discussion section. We already explained that we implicitly impute by accounting for age, sex and education in our model. We now also added that the trend break adjustment also accounts for the impact of changes in missingness over time due to the trend breaks.

The model has some limitations with regard to the data on which the model was fit. The data contains missing values, and these records were removed for fitting each submodel. From 2010 until 2013 there was a relatively large group of missing values in the alcohol items of the survey (+/-45%) in contrast to the other years (<5%). As our model already accounts for a trend break in this period, and accounts for (and thus imputes based on) age, sex and education, we did not further impute these missing data. This may induce a bias to the model if the missing values are not related to age, sex or education.

*- These models include demographics as the predictors of the alcohol consumption. I would like the ask if authors think that the changes in alcohol policy would influence the alcohol consumption. As for future studies, is it possible to incorporate modifiable factors or the factors that can be influenced by alcohol policy in the models?*

Our model is suitable to implement prevention policies by adding their effect on -in the end- the NABW. The effects of alcohol policies are generally measured through a change in the number of alcoholic beverages per week (NABW), or grams of alcohol (Fellbaum et al., 2023; Mun et al., 2023; Platt et al., 2016; Tanner-Smith & Risser, 2016). Hence, we chose to only include the NABW as a modifiable factor. Notably, the NABW effect can be specified by age, sex and education in our model. If behavior change due to an intervention is expected to change within individuals over time, an initial and a preserved NABW effect can also be presumed. In the end, a health simulation model would serve to compare their scenarios to the modelled current practice scenario reflecting status quo.

For the second question: it would be interesting to include other modifiable factors in our model besides the NABW, such as determinants on behavior or on socio-economic context. However, other data sources may be required (e.g. longitudinal, or social contact data), making the model increasingly complex. Also, it will be more difficult to eventually make future forecasts in health simulation models for these variables.

An improved modifiable factor could be implemented in the heavy drinking model as future research. For heavy drinking, all intervention effects now need to be specified through an effect on the NABW. The alcohol model could be extended by including heavy drinking status *regardless* of NABW consumption. Instead of modelling heavy drinking as a dichotomous variable, it can also be modelled from the survey data as a count variable indicating how many times an individual has drunk more than 4 (women) or 6 (men) glasses of alcohol (“higher-consumption days”). The NABW would then have to be made up as a sum of this variable, and of the remaining alcohol consumption on “lower-consumption” days. Then, intervention effects on heavy drinking, regardless of the level of NABW consumption, can be modelled by specifying transitions from drinking alcohol on “high-consumption” occasions to “low-consumption” occasions. This is now added to the discussion.

Heavy drinking behaviour can be modelled more explicitly by recording the number of occasions per week that an individual exceeds the heavy drinking threshold, rather than our current practise of modelling heavy drinking as a dichotomous variable. This would make it possible to incorporate interventions on heavy drinking behaviour, regardless of NABW consumption.

*- Regarding the generalizability of the study, please give us insights on whether this model would be able to be used outside of the Netherlands. If it is possible, please give some details on the potential conditions.*

It indeed is important to share our thoughts on the generalizability of the study. The model is fitted to Dutch data, but it can be extended to other countries, where similar data often is available to re-estimate the model equations. We now explain that/how the model can be used outside the Netherlands in the last paragraph of the discussion.

We constructed an model to predict alcohol consumption patterns based on Dutch data. The model can be generalized by fitting (elements of) the model to health survey data from other countries, being generally available (WHO, 2025). Although countries may use different terminology and definitions for their alcohol consumption patterns, the model can be applied for similar indicators, notably by using different cut-off values for excessive or heavy episodic drinking.

# References

Boer, J. K., T

Edens, J, Ekelenboom, M., Nawijn, E., Koopman, N., de Pon, E., Pees, S., Hendriksen, M., & van Giessen, A. (2022). *Voortgangsrapportage Nationaal Preventieakkoord 2021*.

Boonstra, H. J. (2025). *Weging gezondheidsenquête, vanaf 2014*. <https://www.cbs.nl/nl-nl/achtergrond/2025/06/weging-gezondheidsenquete-vanaf-2014>

Brennan, A., Meier, P., Purshouse, R., Rafia, R., Meng, Y., Hill-Macmanus, D., Angus, C., & Holmes, J. (2015). The Sheffield alcohol policy model - A mathematical description [Article]. *Health Economics (United Kingdom)*, *24*(10), 1368–1388. <https://doi.org/10.1002/hec.3105>

Britton, A., Ben-Shlomo, Y., Benzeval, M., Kuh, D., & Bell, S. (2015). Life course trajectories of alcohol consumption in the United Kingdom using longitudinal data from nine cohort studies. *BMC Med*, *13*, 47. <https://doi.org/10.1186/s12916-015-0273-z>

Bruggink, J.-W., & van Herten, M. (2014). *Weging Gezondheidsenquête, vanaf 2010*. <https://www.cbs.nl/nl-nl/onze-diensten/methoden/onderzoeksomschrijvingen/korte-onderzoeksomschrijvingen/gezondheidsenquete-vanaf-2010-2013>

CBS. (2005). *Permanent Onderzoek Leefsituatie*. <https://www.cbs.nl/nl-nl/onze-diensten/methoden/onderzoeksomschrijvingen/korte-onderzoeksomschrijvingen/gezondheidsenquete-1981-1996-en-pols-gezondheid-1997-2009>

CBS. (2025a). *Gezondheidsenquête 1981-1996 en POLS-Gezondheid 1997-2009*. Centraal Bureau voor de Statistiek (CBS) / Statistics Netherlands. <https://www.cbs.nl/nl-nl/onze-diensten/methoden/onderzoeksomschrijvingen/korte-onderzoeksomschrijvingen/gezondheidsenquete-1981-1996-en-pols-gezondheid-1997-2009>

CBS. (2025b). *Gezondheidsenquête vanaf 2010-2013*. Centraal Bureau voor de Statistiek (CBS) / Statistics Netherlands. <https://www.cbs.nl/nl-nl/onze-diensten/methoden/onderzoeksomschrijvingen/korte-onderzoeksomschrijvingen/gezondheidsenquete-vanaf-2010-2013>

CBS. (2025c). *Gezondheidsenquête vanaf 2014*. Centraal Bureau voor de Statistiek (CBS) / Statistics Netherlands. <https://www.cbs.nl/nl-nl/onze-diensten/methoden/onderzoeksomschrijvingen/korte-onderzoeksomschrijvingen/gezondheidsenquete-vanaf-2014>

Cobben, F., Roels, J., Wong, F. Y., Buelens, B., Banning, R., van den Brakel, J., & Schouten, B. (2012). *Methodologisch onderzoek bij het herontwerp van de Gezondheidsenquête 2010*.

Eykelenboom, M., Boer, J. M. A., ten Dam, J., Edens, J. J., Kuijpers, T. G., Koopman, N., Sanderman-Nawijn, E. L., Blokstra, A., & van Giessen, A. (2024). Doorrekening impact Nationaal Preventieakkoord: deelakkoord problematisch alcoholgebruik. Worden de ambities voor 2040 bereikt? In: Rijksinstituut voor Volksgezondheid en Milieu RIVM.

Fellbaum, L., Mojzisch, A., Bielefeld, L., Benit, N., & Soellner, R. (2023). The effectiveness of workplace interventions for the prevention of alcohol use: A meta-analysis. *Addiction*, *118*(11), 2043–2061. <https://doi.org/https://doi.org/10.1111/add.16276>

Furtwængler, N. A. F. F., & de Visser, R. O. (2013). Lack of international consensus in low-risk drinking guidelines. *Drug and Alcohol Review*, *32*(1), 11–18. <https://doi.org/https://doi.org/10.1111/j.1465-3362.2012.00475.x>

Gezondheidsraad. (2015). *Alcohol - Achtergronddocument bij bij Richtlijnen goede voeding 2015*.

Koenig, L. B., Haber, J. R., & Jacob, T. (2020). Transitions in alcohol use over time: a survival analysis. *BMC Psychology*, *8*(1), 115. <https://doi.org/10.1186/s40359-020-00479-1>

Morren, M., & Willems, R. (2015). *Gezondheidsenquête 2014: een analyse van de methodebreuk*.

Mun, E.-Y., Zhou, Z., Huh, D., Tan, L., Li, D., Tanner-Smith, E. E., Walters, S. T., & Larimer, M. E. (2023). Brief Alcohol Interventions are Effective through 6 Months: Findings from Marginalized Zero-inflated Poisson and Negative Binomial Models in a Two-step IPD Meta-analysis. *Prevention Science*, *24*(8), 1608–1621. <https://doi.org/10.1007/s11121-022-01420-1>

OECD. (2024). *SPHeP-NCDs Technical Documentation*. Organisation for Economic Co-operation and Development (OECD). <http://oecdpublichealthexplorer.org/ncd-doc>

Platt, L., Melendez-Torres, G. J., Donnell, A., Bradley, J., Newbury-Birch, D., Kaner, E., & Ashton, C. (2016). How effective are brief interventions in reducing alcohol consumption: do the setting, practitioner group and content matter? Findings from a systematic review and metaregression analysis. *BMJ Open*, *6*(8), e011473. <https://doi.org/10.1136/bmjopen-2016-011473>

Probst, C., Buckley, C., Lasserre, A. M., Kerr, W. C., Mulia, N., Puka, K., Purshouse, R. C., Ye, Y., & Rehm, J. (2023). Simulation of Alcohol Control Policies for Health Equity (SIMAH) Project: Study Design and First Results. *Am J Epidemiol*, *192*(5), 690–702. <https://doi.org/10.1093/aje/kwad018>

Purshouse, R. C., Meier, P. S., Brennan, A., Taylor, K. B., & Rafia, R. (2010). Estimated effect of alcohol pricing policies on health and health economic outcomes in England: an epidemiological model [Article]. *The Lancet*, *375*(9723), 1355–1364. <https://doi.org/10.1016/S0140-6736(10)60058-X>

R Core Team. (2024). R: A language and environment for statistical computing. R

Foundation for Statistical Computing, Vienna, Austria.

Tanner-Smith, E. E., & Risser, M. D. (2016). A meta-analysis of brief alcohol interventions for adolescents and young adults: variability in effects across alcohol measures. *Am J Drug Alcohol Abuse*, *42*(2), 140–151. <https://doi.org/10.3109/00952990.2015.1136638>

VZInfo. (2024). *Alcoholgebruik | Ziektelast*. Retrieved August 12th, 2025 from <https://www.vzinfo.nl/alcoholgebruik/ziektelast>

WHO. (2025). *The Global Health Observatory - Global Information System on Alcohol and Health*. World Health Organization (WHO). <https://www.who.int/data/gho/data/themes/global-information-system-on-alcohol-and-health>

Wong, F. Y., Roels, J., & van den Brakel, J. (2011). *Analyse methodebreuken Gezondheidsenquête bij het herontwerp Persoonsenquête*.
